# Kruppel like factor 16 promotes lung adenocarcinoma progression by upregulating lamin B2

**DOI:** 10.1080/21655979.2022.2060780

**Published:** 2022-04-07

**Authors:** Xiaodan Jiao, Weinian Gao, Hongxin Ren, Yanning Wu, Tiezhi Li, Shujun Li, Hongjiang Yan

**Affiliations:** a Department of Respiratory Medicine, The Second Hospital of Hebei Medical University, Shijiazhuang, Hebei, China; b Department of Cardiac Surgery, The Second Hospital of Hebei Medical University, Shijiazhuang, Hebei, China; c Department of Internal Medicine, Yuanshi County Hospital, Yunshi, Jiangsu, China; d Department of Infectious Disease, The Second Hospital of Hebei Medical University, Shijiazhuang, Hebei, China; e Department of Thoracic Surgery, The Second Hospital of Hebei Medical University, Shijiazhuang, Hebei, China

**Keywords:** Lung cancer, KLF16, LMNB2, cancer biomarker, prognosis

## Abstract

Lung cancer is one of the most common causes of cancer-related death. In the past decade, the treatment and diagnosis of lung cancer have progressed significantly in early efforts to promote the survival of lung cancer patients. Kruppel like factor 16 (KLF16) is a zinc finger transcription factor that regulates a diverse array of developmental events and cellular processes. KLF16 is involved in the progression of various cancer types. However, the role of KLF16 in the development of lung cancer remains unknown. In this study, KLF16 was overexpressed in lung cancer samples. KLF16 downregulation inhibited lung cancer cell proliferation and migration. Conversely, KLF16 overexpression promoted lung cancer cell growth and invasion. Mechanistically, the expression level LMNB2 was suppressed by KLF16 knockdown and was promoted by KLF16 overexpression. The overall survival of patients with high LMNB2 levels was poor. Luciferase assays showed that KLF16 promoted the transcription activity of LMNB2 gene. Concomitantly, the expression level of LMNB2 was also higher in lung adenocarcinoma (LUAD) than in normal tissues, and its knockdown or overexpression can reverse the effect of KLF16 overexpression or knockdown on lung cancer cell proliferation, migration, and even tumorigenesis, indicating that LMNB2 also functions as an oncogene. In conclusion, KLF16 can be used as a potential therapeutic and preventive biomarker in lung cancer treatment and prognosis by actively regulating the expression of LMNB2.

## GRAPHICAL ABSTRACT

**Figure:**
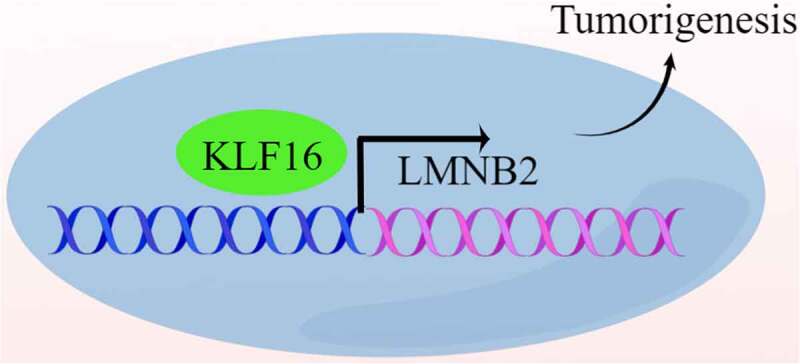

## Introduction

Lung cancer is the third most common cancer worldwide. Approximately 90% of lung cancer cases are caused by smoking, and long-term exposure to cigarettes causes dysplasia of the lung epithelium and cell cycle damage, which promotes carcinogenesis [1]. Lung cancer can be classified histologically into two categories: small cell lung carcinoma (SCLC, 15% of lung cancer cases) and non-small cell lung cancer (NSCLC, 85% of lung cancer cases). NSCLC is usually subdivided into three types: adenocarcinoma, squamous cell carcinoma, and large cell carcinoma [2]. The accurate identification of lung cancer staging based on the characteristics of primary tumor (T), regional nodes (N), and metastatic disease (M) is important for determining the appropriate treatment method [3]. The current treatments for lung cancer include surgery, chemotherapy, radiotherapy, and immunotherapy [4]. Blood biomarkers, autoantibodies, microRNAs (miRNAs), circulating tumor DNA, exosomes, and other possible biomarkers (DNA methylation and polymorphisms) have been used for determining the diagnosis and prognosis of lung cancer [5]. In the past decade, significant progress has been made in the treatment and diagnosis of lung cancer; the main improvements were aimed at improving the overall survival [6]. Despite all of these advances, the outcomes for lung cancer remains unoptimistic, and the 5-year survival rate is still >18%[7]. Hence, new treatment methods or biomarkers must be developed and identified to improve the survival rate of patients with lung cancer.

Recently, several studies have reported the involvement of several important biomarkers in the development of lung cancer [8–10]. Krüppel-like factors (KLFs) are zinc finger transcription factors that regulate a diverse array of developmental events and cellular processes by activating and/or inhibiting the expression of a large number of genes [11]. Currently, 17 KLFs have been annotated in the human genome, which can be divided into two categories: KLF1, KLF2, KLF4, KLF5, KLF6, and KLF7 are activators, whereas the others are repressors [12]. KLF16, also known as BTEB4 or NSLP2, is an important regulator involving in the progression of myocardial ischemia-reperfusion [13]. Besides, dysregulation of KLF16 has also been reported to play a critical role in the development of several types of cancer such as prostate cancer [14], breast cancer [15], gastric cancer [16], pancreatic cancer [17], and anaplastic thyroid carcinoma [18]. In addition, KLF16 overexpression contributes to the malignant growth of retinoblastoma cells through positively regulating the expression of CRABP2 [19]. These results suggest that KLF16 is a potential biomarker for the treatment and prevention of human cancers. However, no study has reported the KLF16 expression in lung cancer.

In this study, we hypothesized that KLF16 might act as an oncogene in the development of lung cancer. This study aimed to explore the function and molecular mechanism of KLF16 in lung cancer development.

## Materials and methods

### Patient samples and immunohistochemistry

A total of 54 patients (25 men and 29 women; mean age 54.2 ± 13.23 years) were recruited from The Second Hospital of Hebei Medical University between March 2018 and July 2019. Cancer tissues were obtained without any treatment intervention. The study was conducted in accordance with the Declaration of Helsinki and was approved by the Ethics Committee of the Second Hospital of Hebei Medical University (no. 2020-R599). Written informed consent was obtained from all patients.

KLF16 expression in lung cancer and normal tissues was detected using immunohistochemistry (IHC). Formalin-fixed paraffin-embedded sections (4 μm) were deparaffinized, dehydrated, and subjected to antigen retrieval. IHC staining was performed using the Ventana BenchMark GX IHC kit (Roche, Basel, Switzerland). Primary KLF16 antibody was purchased from Abcam (ab187973, 1:100 dilution). The expression of KLF16 in tissues was quantified using the following signal intensities: 0 (negative), 1 (low), 2 (moderate), and 3 (high). The KLF16 expression was considered as ‘low’ when the signal intensity score was 0 or 1, whereas it was considered as ‘high’ when the score was 2–3 [20].

### The Cancer Genome Atlas analysis

For The Cancer Genome Atlas (TCGA) analysis, the expression levels of KLF16 and LMNB2 were analyzed using the TCGA lung adenocarcinoma dataset downloaded from a website (http://www.sysu.edu.cn/403.html). The correlation between KLF16 and LMNB2 was analyzed using linear regression in the TCGA database (http://gepia.cancer-pku.cn). Kaplan–Meyer analysis was performed using the Gene Expression Profiling Interactive Analysis tool, and the cutoff value was determined (http://gepia.cancer-pku.cn) [21].

### Cell culture and transfection

The human lung cancer cell lines H1299, H1975, and A549 and human normal lung epithelial cells (BEAS-2B) were purchased from the American Type Culture Collection. The cancer cells were cultured in Roswell Park Memorial Institute (RPMI) 1640 (RPMI-1640) medium (Gibco) supplemented with 10% fetal bovine serum and 1% penicillin/streptomycin (Sigma), while the BEAS-2B cells were cultured in serum-free LHC-9 medium. All cells were cultured in a humidified incubator containing 5% CO_2_ at a temperature of 37°C [22].

KLF16 knockdown in H1299 and H1975 cells was performed using the RNAiMAX kit (Invitrogen) [23], according to the manufacturer’s protocol. KLF16 knockdown was performed using a small interfering RNA (siRNA). siRNAs were purchased from Huzhou Hippo Biotechnology (Huzhou, China): siKLF16#1: 5′-GGGUUCUUCCAAAGAACAU-3′; siKLF16#2: 5′-GGUAUCACGUGACAAUCAA-3′; siLMNB2: 5′-GGCTGCAGGAGAAGGAGGAGCTG-3; and siCtrl: 5′-UUCUCCGAACGUGUCACGU-3′. For KLF16 overexpression (NM_031918.4, https://www.ncbi.nlm.nih.gov/nuccore/NM_031918.4) and knockdown in a xenograft model, the cells were infected with lentivirus. The lentiviruses were purchased from GeneChem (Shanghai, China). After selection with puromycin for 7 days, the cells were subjected to overexpression or xenograft model assays.

### Real time quantitative PCR (RT-qPCR) assay

Total RNA was isolated using an RNAiso Plus Reagent (Takara). RNA was transcribed using a PrimeScript RT Reagent Kit (Beyotime Biotechnology). RT-qPCR was performed using SYBR Premix Ex Taq^TM^ II (Takara) [24] on an RT-PCR system (ABI 7500). The qPCR primers used were as follows: 5′-TGGGCAAACCCTGAAGACA-3′ for KLF16-F and 5′-GTTGCACAGATGGGAAGAAA-3′ for KLF16-R, 5-GGCTGCAGGAGAAGGAGGAGCTG-3 for LMNB2-F and 5-CATCACGTAGCAGCCTCTTGAG-3 for LMNB2-R, and 5-CATGTACGTTGCTATCCAGGC-3 for β-actin-F and 5-CTCCTTAATGTCACGCACGAT-3 for β-actin. Gene expression was evaluated using the 2^−ΔΔCt^ method [25].

### Western blotting

Total protein was collected using RIPA lysis buffer (Beyotime Biotechnology), and the protein concentration was evaluated using a bicinchoninic acid kit (Thermo Fisher). A total of 30 μg was used for sodium dodecyl sulfate-polyacrylamide gel electrophoresis and then was transferred to the nitrocellulose membranes. After blocking with 5% nonfat milk, the membranes were incubated with primary antibodies at 4°C overnight. The membranes were then incubated with secondary antibodies at room temperature for 2 h. Finally, the proteins were visualized using an enhanced chemiluminescence detection system (Bio-Rad). The following primary antibodies were used: KLF16 (Invitrogen, PA5-103,504, 1:1,000), LMNB2 (Proteintech, 10,895-1-AP, 1:1,000), and GAPDH (Proteintech, 60,004-1-Ig, 1:1,000) [20].

### Cell proliferation

Cell proliferation was detected using the Cell Counting Kit 8 (CCK-8; Beyotime Biotechnology). One thousand cells were seeded in a 96-well plate and grown at 37°C. The cell viability was detected at 24, 48, 72, and 96 h. Before testing, 10 μL of CCK-8 reagent was added to each well and incubated at 37°C for 2 h. Finally, the OD value at 450 nm was measured using a spectrophotometer [26].

### Colony formation

The transfected H1299 and H1975 cells were seeded in a six-well plate at a density of 1,000 cells/well. The cells were cultured at 37°C, and the culture medium was replaced every 3 days. After 14 days of continuous culture, the medium was discarded and the plates were washed with PBS. The clones were fixed with 4% paraformaldehyde and stained with 0.1% crystal violet for 20 min [27].

### Cell cycle analysis by flow cytometry

The cells were trypsinized, centrifuged at 13,000 rpm for 5 min, and washed with cold PBS. The cells were fixed with 75% ethanol at 4°C overnight. After washed by PBS, the cells were pelleted and stained in 0.5 mL of a staining solution (10 µL propidium iodide solution and 10 µL RNase A) at room temperature in the dark for 30 min. Finally, the cell cycle distribution was analyzed ***by flow cytometry*** using CytoFLEX S (Beckman Coulter) [22].

### Apoptosis analysis by flow cytometry

Cell apoptosis was assessed using an Annexin V-FITC/PI Apoptosis Detection Kit (Yeasen, Shanghai, China). Briefly, the H1299 and H1975 cells were harvested using trypsin and rinsed with PBS. The cells were resuspended in buffer and incubated with annexin V-FITC and PI. Apoptosis was measured ***by flow cytometry*** using CytoFLEX S (Beckman Coulter). The percentage of apoptotic cells was calculated as the percentage of early and late apoptotic cells [28].

### Transwell assay

A total of 150 μL of cell suspension (3.0 × 10^4^) in serum-free medium was seeded into the top Transwell chamber (Corning). Complete medium (500 µL; RPMI-1640 medium with 10% fetal bovine serum) was added to the lower chamber. After 48 h of culture, the non-migrated cells were removed, while the migrated cells were fixed and stained with 0.5% crystal violet [29].

### Luciferase assay

To examine the effects of KLF16 on LMNB2 transcription, pGL3 plasmids containing a firefly reporter were used to construct recombinant plasmids with the LMNB2 promoter. Recombinant LMNB2 promoter plasmids combined with the Ctrl or KLF16 overexpression plasmid were co-transfected with pRLTK Renilla in BEAS-2B cells using Lipofectamine 2000 (Invitrogen). In addition, siRNAs against negative control or KLF16 were co-transfected with pGL3-LMNB2 and pRLTK Renilla in H1299 and H1975 cells by Lipofectamine 2000 (Invitrogen). After 48 h of transfection, the luciferase activity was measured using a dual-luciferase reporter assay system (Promega) [13].

### Caspase-3/7 activity assay

Caspase-3/7 activation assay was performed using the Caspase-Glo 3/7 assay kit (Promega Corporation), according to the manufacturer’s instructions. In brief, a total of 1 × 10^4^ cells per well were plated in 96-well plates, and the cells were transfected with siKLF16#1/2 or KLF16-overexpressing vectors for 48 h. Then, 100 μL of Caspase-Glo 3/7 reagent was added to each well, the plates were incubated at room temperature for 2 h, and luminescence was measured using a SpectraMax Microplate Luminometer (Molecular Devices, LLC) [30].

### Xenograft model

Male BALB/c mice (5 weeks old) were purchased from Charles River (Beijing, China). All nude mice were housed in a specific pathogen-free laboratory, and the experiments were performed between January 2021 and February 2021. The H1299 cells were infected with control vector (Ctrl), KLF16 overexpression (KLF16), or KLF16 overexpression combined with LMNB2 knockdown (KLF16+ shLMNB2); cultured; and prepared for injection (100 μL, 2 × 10^6^ cells per mouse). The mice were randomly divided into three groups. The cells were resuspended and injected subcutaneously in the flank of each nude mouse. Thirty days later, the mice were sacrificed using carbon dioxide (CO_2_); the CO_2_ flow rate was 10%–30% of the cage volume per minute, according to previous studies [31], and the tumor weight was measured. This study was approved by the Animal Ethics Committee of the Second Hospital of the Hebei Medical University [29].

### Statistical analysis

The data were expressed as mean ± standard deviation. Student’s *t*-test was used to assess the differences between two groups, while one-way analysis of variance followed by Tukey’s multiple-comparison test was used to assess differences among three or four groups. All statistical analyses were performed using the GraphPad Prism 8.0. A *P*-value <0.05 was considered significant.

## Results

We suspected that KLF16 acted as an oncogene in lung cancer development. Thus, the present study aimed to investigate the clinical significance, cellular function, and downstream effector of KLF16 in lung cancer. We analyzed the expression of KLF16 based on TCGA database and IHC staining. Gain-of-function and loss-of-function experiments were performed to explore the *in vitro* and *in vivo* function of KLF16 in lung cancer cells. Furthermore, qRT-PCR, immunoblotting, luciferase reporter and rescue assays were carried out to investigate the relationship between KLF16 and LMNB2 in lung cancer cell growth, migration and tumorigenesis.

### Upregulation of KLF16 in lung cancer

**Figure 1.**
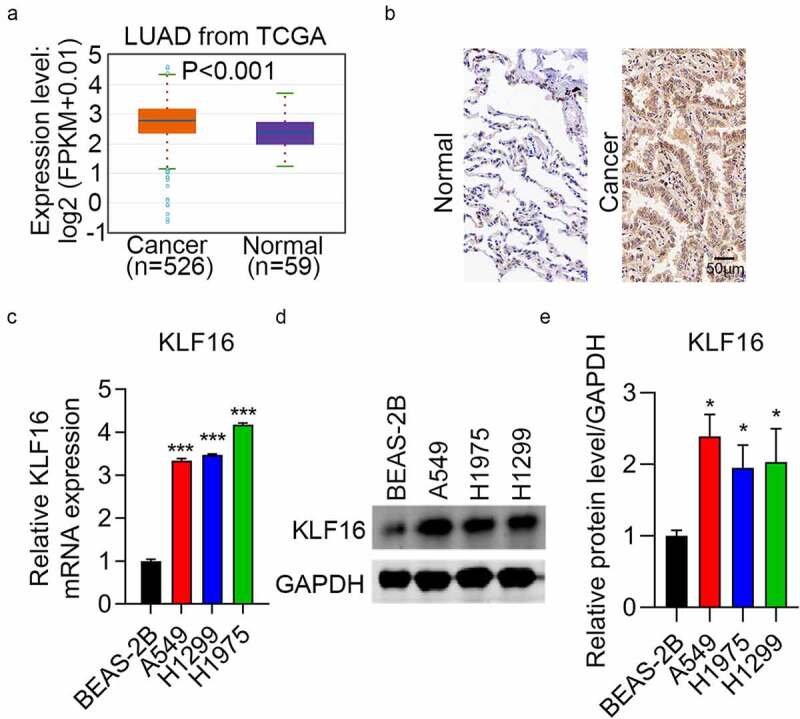
Upregulation of KLF16 in lung cancer samples. (a) KLF16 expression in lung adenocarcinoma (LUAD, *n* = 526) and normal samples (*n* = 59) from The Cancer Genome Atlas. (b) Immunohistochemical staining results of KLF16 expression in LUAD and normal tissues. (c, d) RT-qPCR (c) and western blotting (d) analysis of KLF16 expression in normal (BEAS-2B) and three lung cancer cell lines (H1299, H1975, and A549). (e) The densitometric analysis corresponding to the western blotting. *P < 0.05; ***P < 0.001.

To identify the role of KLF16 in lung cancer, data from TCGA database (526 cancer samples and 59 normal samples) were analyzed. The expression of KLF16 was higher in LUAD than in normal samples (Figure 1(a)). Immunohistochemical staining confirmed this result (Figure 1(b) and Table 1). Furthermore, RT-qPCR and western blotting revealed that KLF16 mRNA and protein levels were higher in three lung cancer cell lines than in normal cells (Figure 1(c-e)). Thus, KLF16 might act as an oncogene.

**Table 1.** The expression of KLF16 in lung cancer and normal tissues

Type	KLF16 expression		χ^2^	P-value
	Low	High		
Normal	39	15	27.08	<0.001
Lung cancer	12	42

### Knockdown of KLF16 inhibiting cell proliferation and migration

**Figure 2.**
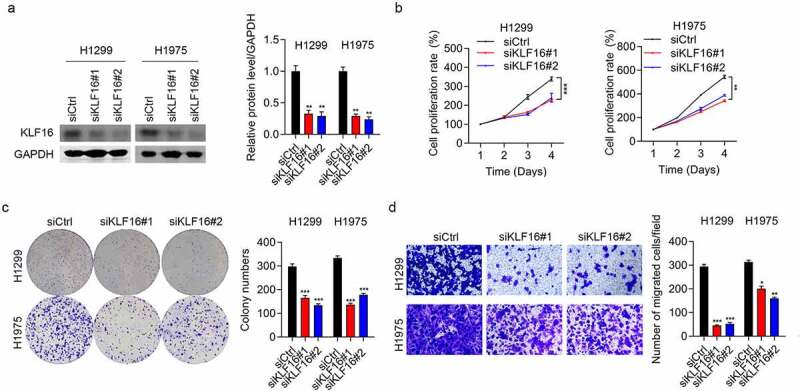
KLF16 knockdown inhibited the cell proliferation and migration. (a) Results of the western blotting of KLF16 levels in H1299 and H1975 cells transfected with siCtrl, siKLF16#1, and siKLF16#2. The loading control used was GAPDH. (b) Results of the CCK-8 analysis of the viability of H1299 and H1975 cells transfected with siCtrl, siKLF16#1, and siKLF16#2. (c) Results of the colony formation assay of H1299 and H1975 cells transfected with siCtrl, siKLF16#1, and siKLF16#2. (d) Results of the Transwell invasion analysis of H1299 and H1975 cells transfected with siCtrl, siKLF16#1, and siKLF16#2. *P < 0.05; **P < 0.01; ***P < 0.001.

siRNAs were used to downregulate the expression of KLF16 in H1299 and H1975 cells. Western blotting and qRT-PCR results showed low KLF16 levels in the siKLF16-transfected cells (Figure 2(a) and Supplementary Figure 1). The cell viability in H1299 and H1975 cells with depletion of KLF16 were significantly decreased (Figure 2(b)). Colony formation in siKLF16-transfected cells was similarly decreased (Figure 2(c)). Moreover, the percentage of migrated cells in H1299 and H1975 lines with KLF16 depletion were also significantly decreased (Figure 2(d)). These findings show that KLF16 depletion can severely inhibit the cell cycle progression, thus suggesting an oncogenic role of KLF16 in lung cancer.

### Overexpression of KLF16 promoting cell proliferation and migration

**Figure 3.**
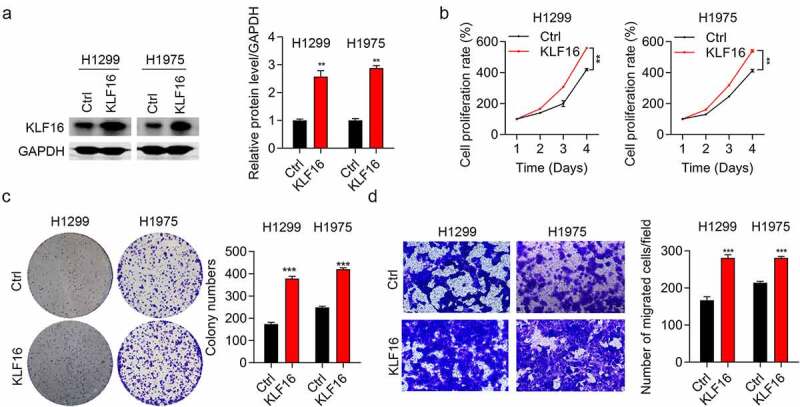
Overexpression of KLF16 promoted cell proliferation and migration. (a) Results of the western blotting of KLF16 levels of H1299 and H1975 cells treated with Ctrl and KLF16. The loading control was GAPDH. (b) Results of the CCK-8 analysis of the cell proliferation of H1299 and H1975 cells treated with Ctrl and KLF16. (c) Results of the colony formation assay of H1299 and H1975 cells treated with Ctrl and KLF16. (d) Results of the Transwell invasion analysis of H1299 and H1975 cells treated with Ctrl and KLF16. **P < 0.01; ***P < 0.001.

Western blotting and qRT-PCR assays were performed to confirm the overexpression of KLF16 in both H1299 and H1975 cells (Figure 3(a) and Supplementary Figure 2). KLF16 overexpression significantly increased the viability of both H1299 and H1975 cells, as observed in the CCK-8 assay (Figure 3(b)). In addition, KLF16 overexpression accelerated the proliferation rate of BEAS-2B cells (Supplementary Figure 3). The colony number (Figure 3(c)) and cell migration (Figure 3(d)) in the KLF16-overexpressing groups were significantly increased in both H1299 and H1975 cells, which was in accordance with our knockdown results.

### KLF16 regulates cell apoptosis and cell cycle

**Figure 4.**
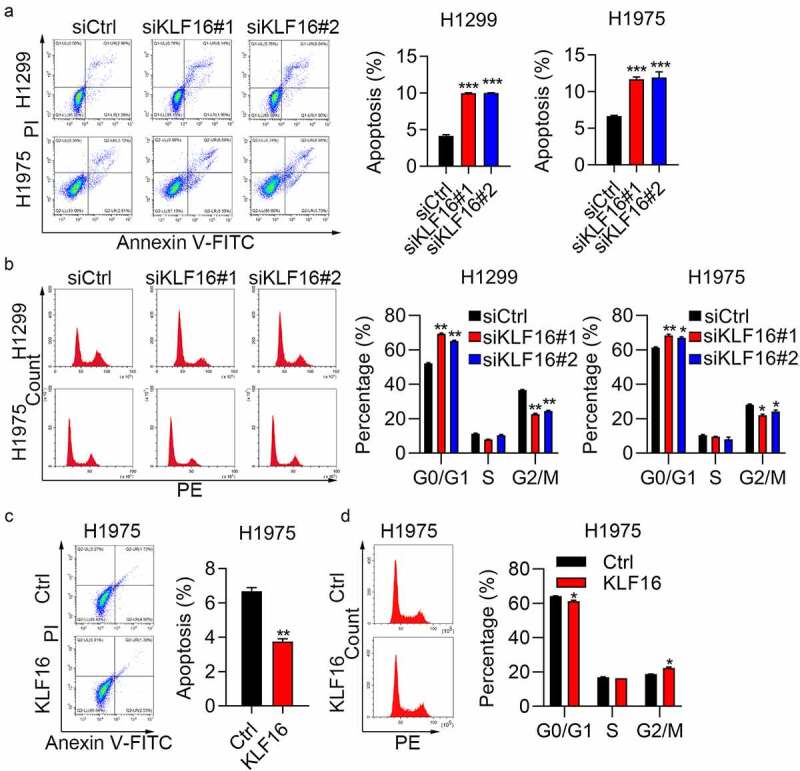
KLF16 regulated cell apoptosis and cell cycle. (a) Cell apoptosis of H1299 and H1975 cells transfected with siCtrl, siKLF16#1, and siKLF16#2 assessed by flow cytometry. (b) Cell cycle of H1299 and H1975 cells transfected with siCtrl, siKLF16#1, and siKLF16#2 assessed by flow cytometry. (c) Cell apoptosis of H1975 cells treated with Ctrl and KLF16. (d) Cell cycle of H1975 cells treated with Ctrl and KLF16. *P < 0.05; **P < 0.01; ***P < 0.001.

Flow cytometry revealed that the percentage of apoptosis in the siKLF16 group was nearly double compared with that in the siCtrl group (Figure 4(a)). In addition, a Caspase-3/7 activity assay was used for caspase 3/7 activity detection; results showed that the activity of caspase 3/7 was significantly increased after transfection with si-KLF16#1/2 in both H1299 and H1975 cells, whereas it was downregulated in KLF16-overexpressing cells (Supplementary Figure 4). KLF16 depletion promoted cell cycle progression, with a decrease in the number of cells in the G2/M phase and an increase in the number of cells in the G0/G1 phase (Figure 4(b)). The overexpression results were also consistent with the knockdown results. Cell apoptosis was inhibited by KLF16 overexpression in H1975 cells (Figure 4(c)). The number of cells in the G2/M stage of the cell cycle increased, while the number of cells in the G0/G1 phase decreased (Figure 4(d)). These results also show that KLF16 functions as an oncogene that regulates cell cycle and apoptosis.

### KLF16 regulates LMNB2 expression

**Figure 5.**
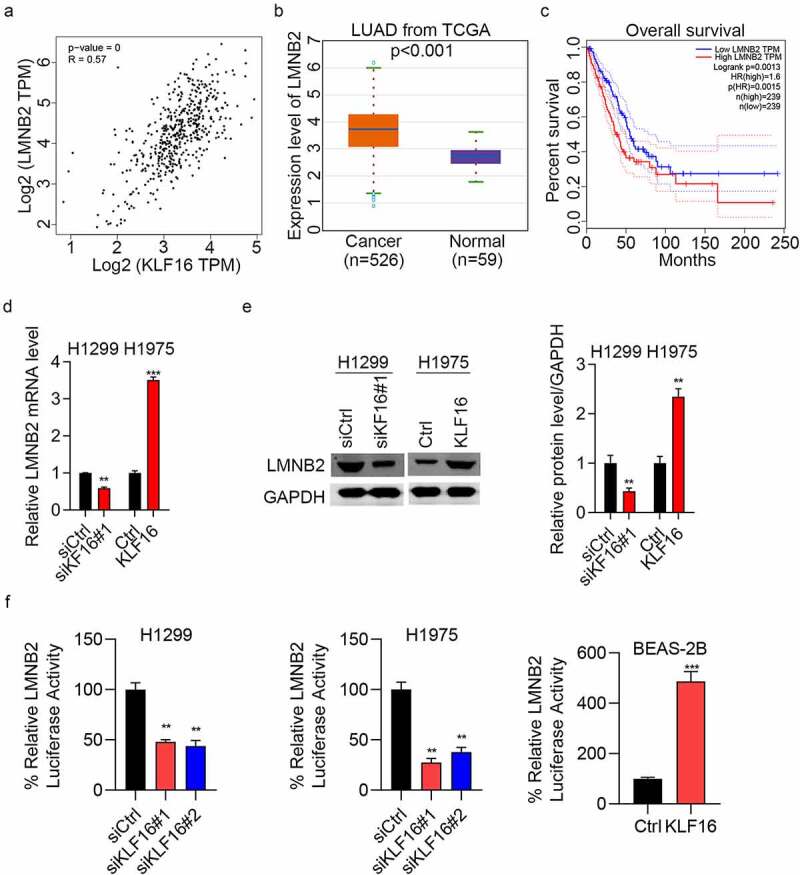
KLF16 regulates the expression of LMNB2. (a) Correlation between KLF16 and LMNB2 expression (R = 0.57, *P* < 0.001) in The Cancer Genome Atlas database. (b) LMNB2 expression in lung adenocarcinoma (n = 526) and normal tissues (n = 59). (c) Overall survival of patients with high LMNB2 and low LMNB2. (d) RT-qPCR of LMNB2 expression in siKLF16-transfected H1299 cells or KLF16-transfected H1975 cells. (e) Western blotting of LMNB2 expression in siKLF16-transfected H1299 cells or KLF16-transfected H1975 cells. The loading control used was GAPDH. (f) siCtrl or siKLF16 was co-transfected with pGL3-LMNB2 and pRLTK Renilla in H1299 and H1975 cells. Ctrl or KLF16 expressing vectors were co-transfected with pGL3-LMNB2 and pRLTK Renilla in BEAS-2B cells. Dual luciferase activity was checked by using Dual-Luciferase Reporter Assay System. **P < 0.01; ***P < 0.001.

The correlation analysis showed that the expression of LMNB2 was positively correlated with the expression of KLF16 (Figure 5(a)). The expression level of LMNB2 in lung adenocarcinoma (LUAD) was measured, and results showed that LMNB2 was highly expressed in LUAD tissues compared with that in normal tissues (Figure 5(b)). Furthermore, the overall survival of patients with high LMNB2 expression was poor (Figure 5(c)). RT-qPCR results showed that LMNB2 was downregulated in siKLF16-transfected H1299 cells and upregulated in KLF16-transfected H1975 cells (Figure 5(d)). Western blotting showed the same results as those obtained by RT-qPCR (Figure 5(e)). Lastly, we showed that KLF16 knockdown reduced the luciferase activity of LMNB2 promoter in H1299 and H1975 cells, whereas KLF16 overexpression exhibited opposite results in BEAS-2B cells (Figure 5(f)). Taken together, KLF16 positively regulates the transcription activity and the expression of LMNB2 gene.

### KLF16 promotes cell progression by LMNB2

**Figure 6.**
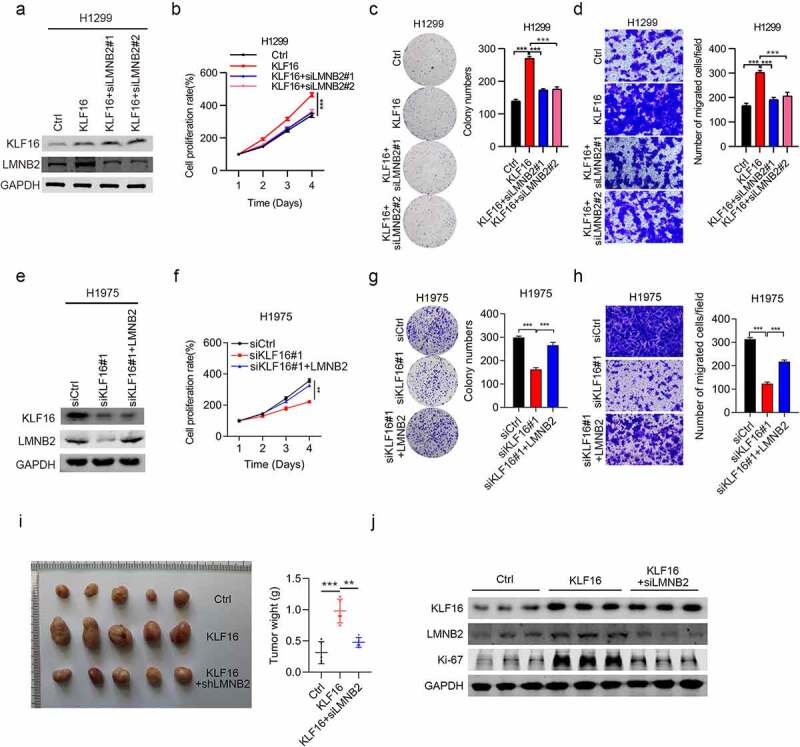
LMNB2 regulates cell progression. (a) Western blotting of KLF16 and LMNB2 expression in H1299 cells treated with Ctrl, KLF16, KLF16 + siLMNB2#1, and KLF16 + siLMNB2#2. (b-d) Cell viability (b), colony formation capacity (c) and cell migration (d) were measured in H1299 cells treated with Ctrl, KLF16, KLF16 + siLMNB2#1, and KLF16 + siLMNB2#2. (e) Western blotting of KLF16 and LMNB2 expression in H1975 cells transfected with siCtrl, siKLF16, and siKLF16 + LMNB2. (f-h) Cell viability (f), colony formation capacity (g) and cell migration (h) were measured in H1975 cells transfected with siCtrl, siKLF16, and siKLF16 + LMNB2. (i) Exogenously expressed Ctrl, KLF16, and KIF16+ shLMNB2 H1299 cells were injected in athymic nude mice (n = 5), and tumor formation was analyzed. Representative images and weight of xenografted tumors are shown. (j) Western blotting of KLF16 and LMNB2 expression in three xenografted tumor tissues. **P < 0.01; ***P < 0.001.

Next, we investigated whether KLF16 promoted lung cancer progression through LMNB2. To address this question, we knocked down LMNB2 in KLF16 overexpressing cells and overexpressed LMNB2 in KLF16 knockdown cells. Firstly, two siRNAs were used to silence LMNB2 in KLF16 overexpressing H1299 cells. Western blot results showed that LMNB2 siRNAs efficiently reduced the expression of LMNB2, while had no effect on KLF16 (Figure 6(a)). CCK8 and colony formation results showed that when KLF16 overexpression promoted the proliferation and growth of H1299 cells, LMNB2 silencing could partly reverse the effect (Figure 6(b,c)). Similar results were observed in the migration capacity of H1299 cells (Figure 6(d)). Then, we overexpressed LMNB2 in KLF16 knockdown H1975 cells (Figure 6(e)). We showed that LMNB2 overexpression rescued the tumor suppressive function of KLF16 knockdown on cell proliferation, growth and migration in H1975 cells (Figure 6(f-h)). Moreover, the in vivo tumorigenicity was measured. Consistent with the above results, when tumor growth was promoted by KLF16 overexpression, it was nearly restored to control levels by LMNB2 knockdown (Figure 6(i,j)). These results indicate that KLF16 promotes lung cancer development through the upregulation of LMNB2.

## Discussion

Several KLF family members are involved in lung cancer progression. KLF2 plays an important role in normal lung development, erythroid differentiation, T-cell differentiation, migration, and homing [32]. KLF3 and KLF4 inhibit the proliferation, migration, and invasion of lung cancer cells, and induce cell cycle arrest and apoptosis [33,34]. KLF7, KLF8, and KLF9 contribute to the progression of non-small cell lung cancer (NSCLC) and are the targets of some miRNAs [35–37]. KLF11 is critical for increasing the sensitization of lung cancer to radiotherapy [38]. KLF17 increases the expression of p53, p21, and pRB; therefore, it may be a new target for the treatment of NSCLC [39]. However, the exact role of KLF16 in lung cancer remains unclear. Herein, we confirmed that KLF16 is an oncogene that is involved in the development of lung cancer. KLF16 downregulation inhibited the proliferation and migration of cancer cells, decreased the number of G2/M phase cells, and promoted apoptosis in H1299 and H1975 cells. Conversely, the overexpression of KLF16 enhanced the proliferation and migration of H1299 and H1975 cells, reduced cell apoptosis, and promoted G2/M phase cells in H1975 cells. This finding strongly indicates that KLF16 plays an important role in lung cancer development; however, its underlying mechanisms are still unclear.

Since KLF16 acts as an important transcription factor, to explore how KLF16 functions as an oncogene in lung cancer, the potential targets of KLF16 in lung cancer were determined. Initially, we analyzed the most significant genes that were positively correlated with KLF16 in LUAD patients from TCGA database. We also analyzed whether these genes were significantly associated with the patients’ prognosis. Among several targets, the human *LMNB2* gene, which locates in chromosome 19p13.3 [40] and encodes a 68-kDa protein, was a potential target for KLF16 in lung cancer development. The main function of *LMNB2* is to maintain the nuclear skeleton integrity, which is involved in cell proliferation and aging, gene expression, and DNA damage repair by affecting the distribution of the chromosomes [41,42]. Deng et al. suggested that LMNB2, a tumor-related protein, could be a target of miRNAs to inhibit the metastasis of lung cancer cells [43]. Here, a positive correlation was found between KLF16 and LMNB2 in lung cancer tissues. LMNB2 expression was transcriptionally activated by KLF16, indicating that LMNB2 might be regulated by KLF16. A luciferase reporter assay was performed to verify the correlation between LMNB2 and KLF16, and the results showed that KLF16 might transcriptionally regulate LMNB2 expression in lung cancer.

A previous study on A549 and H1299 cells reported that LMNB2 knockdown inhibited colony formation, cell proliferation, and G1/S cell cycle progression and increased the apoptosis [44]. Subsequently, the role of LMNB2 in the development of lung cancer was investigated, and results showed that the depletion of LMNB2 inhibited the proliferation and invasion of H1299 and H1975 cells. These results suggest that LMNB2 acts as an oncogene in lung cancer, which is consistent with the results of previous studies. We also analyzed the expression of LMNB2 and found that LMNB2 was highly expressed in LUAD cells compared with that in normal cells. In addition, the overall survival of patients with high LMNB2 expression levels was relatively poor, indicating that LMNB2 may be a prognostic indicator for lung cancer.

Furthermore, rescue assays were conducted to confirm the correlation between LMNB2 and KLF16, and results demonstrated that knockdown of LMNB2 inhibited the proliferation and migration of H1299 and H1975 cells, which were induced by the overexpression of KLF16 cells. By contrast, LMNB2 overexpression induced the proliferation and migration of both H1299 and H1975 cells, which were inhibited by KLF16 knockdown. These results indicate that KLF16 promotes the progression of lung cancer by regulating LMNB2 expression.

Although our study illustrated a story that KLF16 upregulated LMNB2 to promote the growth, proliferation, migration and tumorigenesis of lung cancer cells, whether other proteins participate in KLF16 driving lung cancer development should be determined in the future.

## Conclusion

In summary, KLF16 acts as an oncogene in lung cancer by regulating the expression of LMNB2. The upregulation of KLF16 and LMNB2 was observed in lung adenocarcinoma samples, which accelerated the proliferation and invasion of H1299 and H1975 cells. The luciferase assay results showed that KLF16 promoted the LMNB2 expression at the transcriptional level. LMNB2 depletion also inhibited the proliferation and invasion of H1299 and H1975 cells. Lung cancer patients with high levels of LMNB2 expression had shorter lifespans. Our study illustrates the mechanism of KLF16 in lung cancer progression-positive regulation of LMNB2 expression and suggests that LMNB2 level may serve as an indicator of prognosis for patients with lung cancer.

## Supplementary Material

Supplemental Material

## Data Availability

All data generated or analyzed during this study are included in this published article.
