# Supplementary material for: Kruppel like factor 16 promotes lung adenocarcinoma progression by upregulating lamin B2
Source: Bioengineered. 2022 Apr 7;13(4):9483–95. doi: 10.1080/21655979.2022.2060780 (PMC9161888; doi:10.1080/21655979.2022.2060780)
Supplement: Supplemental Material [file KBIE_A_2060780_SM6557.docx]

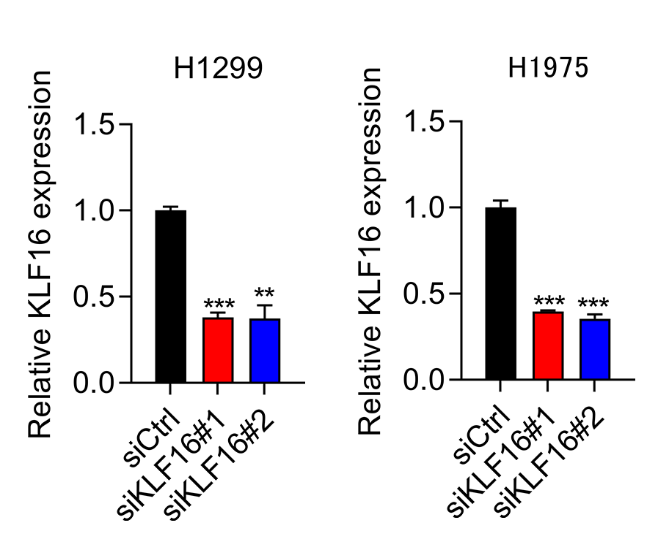


**Supplementary Figure 1.** The relative mRNA expression of KLF16 in cells transfected with siKLF16#1/2 in H1299 and H1975 cells, respectively. ***P* < 0.01; ****P* < 0.001.


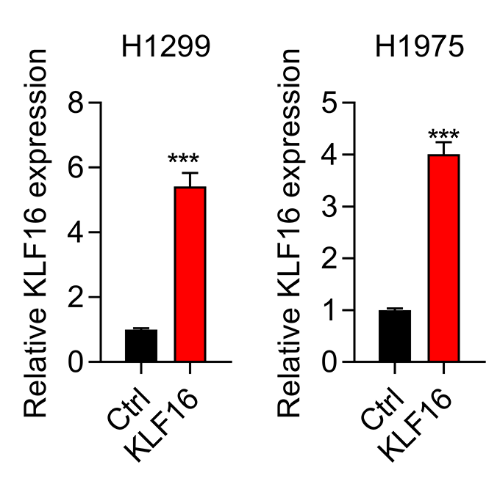


**Supplementary Figure 2.** The relative mRNA expression of KLF16 in cells transfected with ctrl and KLF16-ovexpressing vectors in H1299 and H1975 cells, respectively. ****P* < 0.001.

**
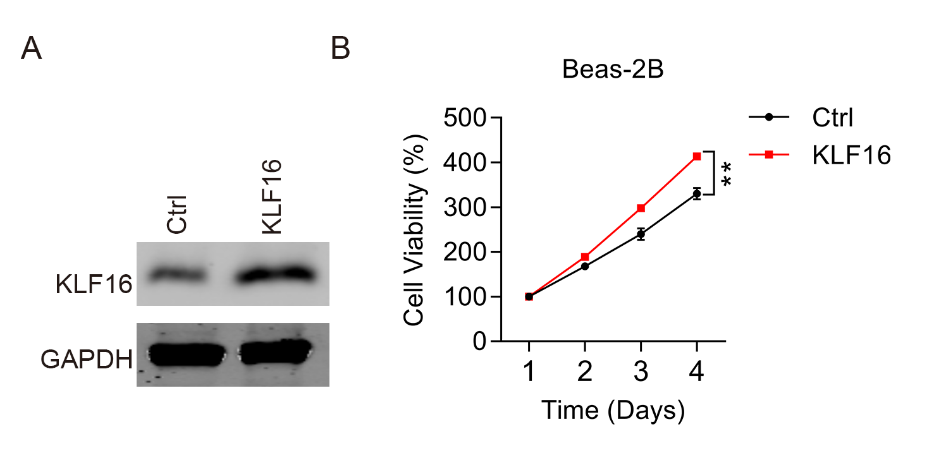
**

**Supplementary Figure 3.** Overexpression of KLF16 promoted cell proliferation of Beas-2B cells. (A) Results of the western blotting of KLF16 levels of Beas-2B cells treated with Ctrl and KLF16. The loading control was GAPDH. (B) Results of the CCK-8 analysis of the cell proliferation of Beas-2B cells treated with Ctrl and KLF16.
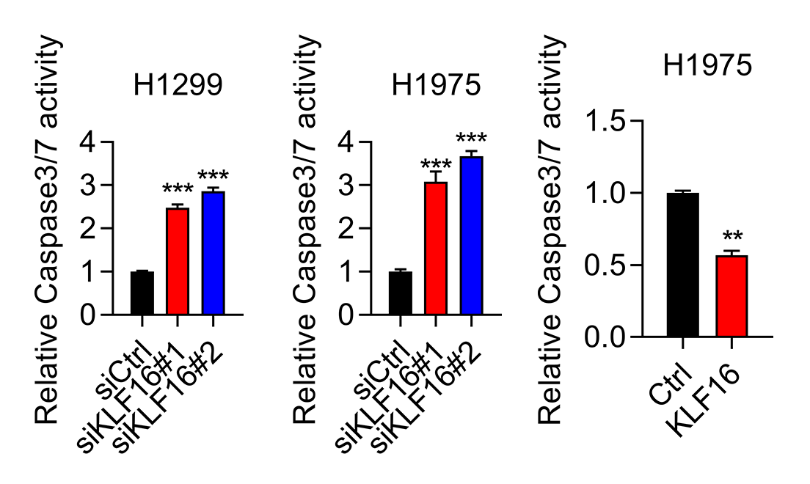


**Supplementary Figure 4.** The relative caspase3/7 activity was determined by Caspase3/7 activation assay in H1299 and H1975 cells, respectively. ***P* < 0.01; ****P* < 0.001.
